# Procalcitonin is an essential biomarker for hydrocortisone, ascorbic acid, and thiamine (HAT) therapy in patients with sepsis

**DOI:** 10.1186/s13054-019-2445-2

**Published:** 2019-05-02

**Authors:** Paul E. Marik

**Affiliations:** Division of Pulmonary and Critical Care Medicine, Eastern Virginia Medical School, 825 Fairfax Av, Suite 410, Norfolk, VA 23507 USA

**Keywords:** Vitamin C, Sepsis, Septic shock, Procalcitonin, Thiamine, Hydrocortisone

**Fig. 1:**
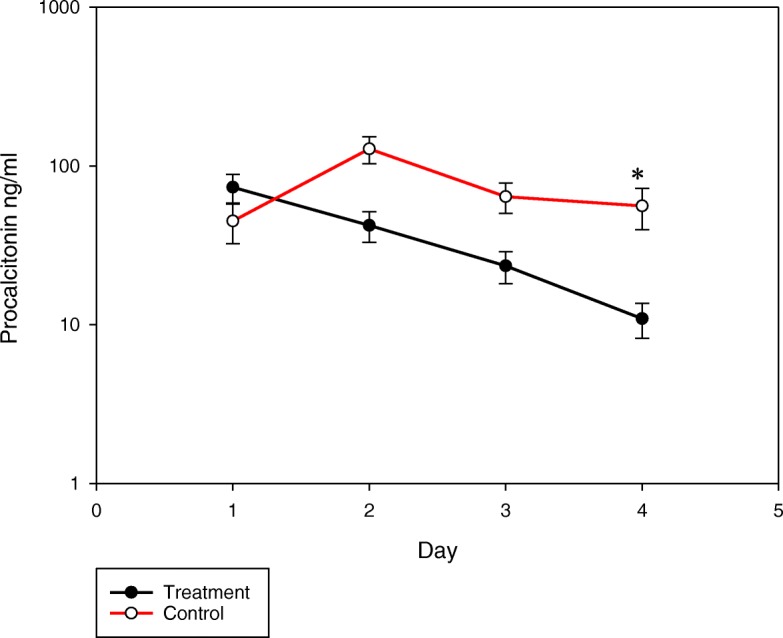
Time course of the serum procalcitonin (PCT) over the 4-day treatment period in the group that received hydrocortisone, ascorbic acid, and thiamine (HAT Rx) as compared to the control group, as reported in our pivotal pilot study [1]. PCT plotted on a semilog scale. *p < 0.001 for comparison of treatment group vs control group. Reproduced with permission from CHEST

There is increasing interest in the use of hydrocortisone, ascorbic acid, and thiamine (HAT Rx) as adjunctive treatment in the management of patients with sepsis and septic shock [1, 2]. We believe that the serial (daily) measurement of procalcitonin (PCT) is an essential component of this strategy. In our pivotal pilot study, we noted that in patients treated with HAT, PCT decreased exponentially [1]. The rate of decline of PCT over 72 h (calculated as the initial PCT minus PCT at 72 h, divided by the initial PCT × 100) was 86% in treated patients compared to 34% in controls (see Fig. 1). The half-life of PCT is reported to be about 24 h [3]; this suggests that with HAT Rx, gene transcription of PCT is switched off, with the decline reflecting the elimination of PCT. Such a rapid decline in PCT has not been reported with any other therapeutic intervention for sepsis [4, 5]. This observation provides biological proof that supports our hypothesis that HAT Rx markedly attenuates the pro-inflammatory response in patients with sepsis, thereby limiting organ failure and improving patient survival [1]. Furthermore, the exponential decline in PCT is not observed with vitamin C alone, when administering vitamin C as a continuous infusion (as a component of HAT Rx) or with alternative dosing strategies (e.g., q 12 hourly dosing of vitamin C). Remarkably, the rapid decline of PCT is even noted in patients with chronic renal failure; this finding being consistent with previous studies [3]. We have now treated over 1200 patients with HAT Rx, and the exponential decline in PCT has been a reproducible finding with few notable exceptions. We have noted that at 24 h after the initiation of therapy, the baseline PCT has failed to fall by 50% in two specific circumstances, namely (i) resistant organism (wrong antibiotic) or (ii) inadequate source control. This is a critical finding as it allows for the early change in antibiotics (broaden spectrum) and/or more aggressive source control. Once these issues are adequately addressed, the PCT then falls in its usual exponential trajectory. Serially monitoring PCT for at least 4 days therefore provides definitive biological proof that HAT therapy is working; failure of the typical PCT trajectory allows early recognition of ongoing inflammation (sepsis) and the need for additional interventions.
